# Hearing impairment in X-linked hypophosphatemia: a review

**DOI:** 10.1093/jbmrpl/ziaf062

**Published:** 2025-12-06

**Authors:** Seongeun Oh, Jessica L Sandy, Craig F Munns, Peter J Simm, Aris Siafarikas, Lucy Collins, Christie-Lee Wall, Maria E Craig, Christine P Rodda, Andrew Biggin

**Affiliations:** Melbourne Medical School, University of Melbourne, Parkville, VIC 3010, Australia; Institute of Endocrinology and Diabetes, The Children’s Hospital at Westmead, Westmead, NSW 2145, Australia; Faculty of Medicine and Health, University of Sydney, Camperdown, NSW 2006, Australia; Child Health Research Centre, Faculty of Medicine, The University of Queensland, Brisbane, QLD, Australia; Department of Endocrinology and Diabetes, Queensland Children’s Hospital, Brisbane, QLD, Australia; Department of Paediatrics, University of Melbourne, Melbourne, VIC 3010, Australia; Department of Endocrinology and Diabetes, Royal Children’s Hospital, Melbourne, VIC 3052, Australia; Centre for Hormone Research, Murdoch Children’s Research Institute, Melbourne, VIC 3002, Australia; Department of Endocrinology and Diabetes, Perth Children’s Hospital, Perth, WA 6009, Australia; Medical School, Paediatrics, University of Western Australia, Perth, WA 6009, Australia; Institute for Health Research, University of Notre Dame, Fremantle, WA 6160, Australia; Western Health, Footscray, VIC 3001, Australia; Institute of Endocrinology and Diabetes, The Children’s Hospital at Westmead, Westmead, NSW 2145, Australia; Institute of Endocrinology and Diabetes, The Children’s Hospital at Westmead, Westmead, NSW 2145, Australia; Faculty of Medicine and Health, University of Sydney, Camperdown, NSW 2006, Australia; Paediatrics & Child Health, School of Clinical Medicine, University of New South Wales, Sydney, NSW 2031, Australia; Department of Paediatrics, University of Melbourne, Melbourne, VIC 3010, Australia; Centre for Hormone Research, Murdoch Children’s Research Institute, Melbourne, VIC 3002, Australia; Institute of Endocrinology and Diabetes, The Children’s Hospital at Westmead, Westmead, NSW 2145, Australia; Faculty of Medicine and Health, University of Sydney, Camperdown, NSW 2006, Australia

**Keywords:** X-linked hypophosphataemic rickets (XLH), hearing impairment, endolymphatic hydrops (ELH), osteomalacia, rickets, deafness

## Abstract

Although hearing impairment is often listed as a nonskeletal complication of X-linked hypophosphatemia (XLH), the prevalence, etiology, pathology, and natural history are poorly described. This review aims to summarize existing literature with a view to guide the clinical management of hearing impairment in XLH. The review was conducted by 2 researchers independently. Four databases (PubMed/Medline, EMBASE, Web of Science, and Cochrane Library) were searched between January 1, 2000 and July 31, 2024, with keywords related to “X-linked hypophosphataemic rickets” and “hearing loss” including synonyms. Identified records were screened for inclusion and exclusion criteria. Human and animal studies were included. Out of 82 records found excluding duplicates, 12 studies met the final criteria and were reviewed. Studies described both conductive and sensorineural hearing loss in 13%-76% of adults with XLH, with sensorineural hearing loss more commonly reported, with impairment developing in adulthood, affecting high and low frequencies, and may be fluctuating. Evidence suggests that endolymphatic hydrops (ELH) may be a major underlying cause of hearing loss in XLH. Individuals with XLH have generalized osteosclerosis with petrous bone thickening and narrowing of the auditory meatus. No studies have looked at burosumab, a monoclonal antibody that inhibits FGF23, and its effect on the development of hearing loss in individuals with XLH. Animal studies of XLH mouse models (*Hyp* and *Gy*) describe both conductive and sensorineural hearing impairment. Mouse models demonstrate high Auditory Brainstem Response (ABR) thresholds and signs of osteomalacia of auditory ossicles and ELH. In conclusion, there is an association between hearing loss in XLH and, most commonly, adult-onset sensorineural hearing loss. Pathogenesis of hearing loss in XLH is incompletely understood, but possible contributing factors include thickening of the temporal bones, osteomalacia of the auditory ossicles, and development of ELH. There is currently no evidence that treatment with conventional therapy or burosumab reduces the risk or severity of hearing impairment.

## Introduction

Hypophosphataemic rickets refers to a group of inherited or acquired disorders characterized by chronic hypophosphatemia due to reduced renal tubular reabsorption of phosphate.^1^ The majority of hypophosphataemic rickets is hereditary, with X-linked hypophosphataemic rickets (XLH) being the most common form, accounting for 80% of all types of rickets, with an estimated prevalence of approximately 1 in 20 000.^2^ XLH has an X-linked dominant pattern of inheritance, where loss-of-function mutations of the *Phosphate Regulating Endopeptidase Homolog X-Linked* (*PHEX*) gene cause inappropriate elevation of serum fibroblast growth factor 23 (FGF23) levels.^1^ In addition to skeletal manifestations, hearing impairment in XLH patients has also been reported in numerous studies since the 1980s.

FGF23 is a bone-derived phosphaturic hormone involved in the downregulation of renal sodium-phosphate co-transporters in the proximal tubules as well as in the inhibition of renal $1\alpha$-hydroxylase and stimulation of $24\alpha$-hydroxylase, causing a reduction in calcitriol levels.^3^ Excess FGF23 level in XLH subsequently leads to a decrease in both renal reabsorption and intestinal absorption of phosphate, resulting in chronic hypophosphatemia. Defective phosphate metabolism contributes to a broad range of local and systemic consequences in affected children and adults.^2^

Historically, the conventional treatment for XLH has been with a combination of phosphate and activated vitamin D supplementation (calcitriol or where this is unavailable 1 alpha hydroxyvitamin D_3_).^4^ In 2018, the development of burosumab, a fully human monoclonal antibody against FGF23, resulted in a ground-breaking transition in the management of XLH. With its specific FGF23 antagonism, more direct normalization of biochemical parameters can be achieved requiring less frequent monitoring for dosing, with considerably less side effects such as secondary/tertiary hyperparathyroidism and nephrocalcinosis.^5^

With regard to hearing impairment, evidence from early key studies largely suggests a predominant sensorineural hearing loss in XLH adults of cochlear origin (similar to that which occurs in Ménière’s disease), rather than neural lesions.^6–9^ These findings are summarized in Table 1. Key cochlear pathology noted across hearing-impaired XLH patients was Endolymphatic Hydrops (ELH), potentially precipitated by generalized structural changes in the petrous bone as noted by O’Malley et al.,^8^ which may alter the endolymph turnover between the stria vascularis and the endolymphatic sac, or cause obstruction of the endolymphatic duct/sac. Multiple potential molecular mechanisms for hearing loss in XLH have been proposed.^1^ These may involve *PHEX* directly, be mediated by increased FGF23, or via other inflammatory processes. Despite these early findings, however, details on the prevalence, etiology, and natural history of hearing loss in XLH remained poorly understood, limited by a small sample size, especially of pediatric patients, and limited technologies. The impact of treatment, either with conventional therapy or FGF-23 inhibition, on hearing impairment is also unclear.

**Table 1:** Key study findings before 2000.

**Author(s)/Year (Reference)**	**Country of origin**	**Study design**	**Population**	**Methodologies**	**Results**
**Davies et al./1984^6^**	England	*Cross-sectional* observational Patients attending the metabolic clinic at the Manchester Royal Infirmary, Manchester	*Human* 25 XLH patients from 16 families Age range: 11-75 yr old 3 Children—11, 13, 15 year old	Pure-tone audiometry Tympanometry Stapedius reflex threshold Caloric testing ^*^Further assessments for those with confirmed sensorineural/mixed hearing loss: Tone decay test Percentage speech discrimination Loud balance test	Nineteen confirmed to have hearing impairment based on combined test results—18 adults, 1 child Sixteen sensorineural, 3 mixed No pure conductive hearing loss was reported One pediatric patient (15 year old male) had sensorineural hearing loss but no subjective symptoms Those with sensorineural hearing loss were confirmed to have a cochlear origin of hearing loss rather than neural lesions Twelve reported subjective hearing loss. Seven reported vertigo, and 8 reported tinnitus Two of these patients met the clinical diagnosis criteria for Ménière’s disease, with signs of ELH
**O’malley et al./1985^7^**	England	*Cross-sectional* observational Patients attending the metabolic clinic at the Manchester Royal Infirmary, Manchester	*Human* Thirteen XLH patients with sensorineural hearing impairment from the patients recruited in the original study by Davies et al. in 1984^4^	Transtympanic electrocochleography	The majority exhibited a cochlear pattern of cochleogram, similar to that in Ménière’s disease Fourteen ears showed results suggestive of Endolymphatic Hydrops (ELH) Proposed ELH as the main cochlear pathology, similar to that which occurs in Ménière’s disease
**O’malley et al./1988^8^**	England	*Cross-sectional* observational Patients attending the metabolic clinic at the Manchester Royal Infirmary, Manchester	*Human* Eleven XLH patients with confirmed hearing impairment Eleven non-XLH control group—patients being investigated for acoustic neuroma at the center	Hypocycloidal tomography on temporal petrous bones bilaterally	Generalized osteosclerosis and thickening of the petrous bone compared to the control group Some degree of narrowing of the internal auditory meatus in its mid-portion compared to the control group Proposed that these structural changes in the petrous bone may contribute to ELH in XLH—either by affecting the endolymph turnover via stria vascularis, or via obstruction of the endolymphatic duct or sac
**Boneh et al./1987^9^**	Canada	*Case-series* Patients attending XLH clinic at the Montreal Children’s Hospital, Quebec	*Human* Twenty-two XLH patients	Pure-tone audiometry Tympanometry Stapedius reflex threshold Tone decay test Speech discrimination	Five patients with sensorineural hearing loss due to cochlear dysfunction All were adults > 20 years

Since the 2000s, more reliable studies have been published with larger adult and pediatric populations, along with the use of more advanced otological methodologies. Screening in older children and adults is most often performed via pure-tone audiometry, which enables the classification of hearing impairment into conductive, sensorineural, or mixed patterns. Other modes of assessment are also often performed, including Auditory Brainstem Response (ABR) thresholds, which reflect the electrical signal of the central auditory pathways and the auditory nerve, as well as Distortion Product Otoacoustic Emission (DPOAE) thresholds, which reflect the outer hair cell function.^10^

Animal studies including numerous mice models of different *PHEX* mutation alleles have been identified, enabling modeling of hearing impairment in human XLH pathogenesis. Widely recognized strains include male mice hemizygous for *Hyp*, *Hyp-Duk*, *Hyp-2J*, and *Gy* alleles, each denoted as *Hyp*/Y, *Phex^Hyp-Duk^*/Y, *Phex^Hyp-2J^*/Y, and *Gy*/Y, respectively.^11^^,^^12^

This review aims to clarify the association between XLH and hearing impairment and to establish the pattern, age of onset, and pathophysiology of hearing impairment in XLH. It is anticipated that these results will guide future clinical management and investigate whether early routine hearing assessment and treatment can effectively delay or improve the degree of hearing impairment in XLH.

## Materials and methods

Databases used: PubMed, EMBASE, Web of Science, Cochrane Library

Search and screening were conducted between November 2023 and July 2024

Keywords: X-linked hypophosphataemic rickets (synonyms X-linked hypophosphataemic rickets, familial hypophosphataemic rickets); hearing loss (synonyms deafness, hearing impairment, deaf, hard of hearing)

Search Query: (((familial hypophosphat^*^) OR (X linked hypophosphat^*^) OR (hereditary hypophosphat^*^) OR (congenital hypophosphat^*^) OR (genetic hypophosphat^*^)) AND ((hearing loss) OR (deafness) OR (hearing impair^*^) OR (deaf) OR (hard of hearing) OR (hearing disorder^*^)))

^*^Filters applied: English language only, published date January 1, 2000–Current.

Inclusion criteria:

– Full-text articles
– English only
– Published date January 1, 2000 to July 31, 2024
– Case series/reports, observational studies (cohort, case–control, cross-sectional studies)
– Human & animal studies

Exclusion criteria:

– Reviews, conference abstracts, notes, comments, book chapter, surveys
– Non-X-linked familial hypophosphataemic rickets, Vitamin D-deficient rickets

## Results

**Figure 1:**
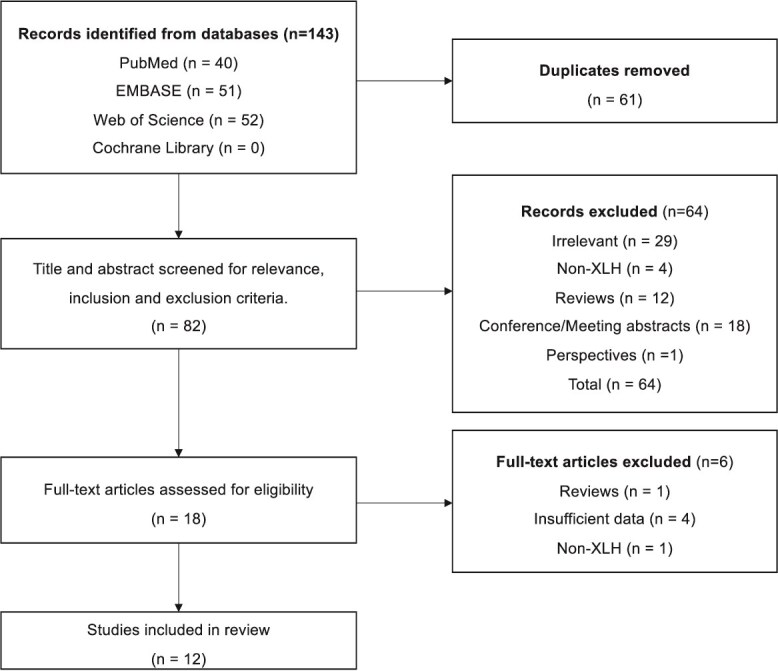
PRISMA diagram.

The search process is outlined as a PRISMA diagram in Figure 1. Twelve studies were selected for the review. The summary of key study characteristics for the selected records is presented in Table 2 for human studies and Table 3 for animal studies. Note that the study by Delsmann et al.^13^ consisted of both human and animal studies hence has been included in both tables.

**Table 2:** Human studies and case series/reports—key study characteristics, results, and limitations.

**Author(s)/Year** **(Reference)**	**Country of origin**	**Study design**	**Population**	**Methodology**	**Results**	**Limitations**
**Delsmann et al./2021^13^**	Germany	*Cross-sectional* observational Patients attending the specialized outpatient clinic at the University Medical Center Hamburg-Eppendorf	*Human* Seven adult female XLH patients (Age 24-53)	Pure-tone audiometry ^*^Patients with conductive hearing loss: Cranial Computed Tomography (CCT) scan of the auditory ossicles to obtain average Hounsfield units (HU) of the auditory ossicles Control: CCT scans of 3 age and sex-matched anonymized patients at the clinic postconcussion Transiliac crest biopsy with Quantitative backscattered electron Imaging (qBEI) and histomorphometric analysis Control: Biopsies from 4 age and sex-matched skeletally healthy cadevars	**Audiometry** Five patients reported subjective hearing loss, 3 patients had sensorineural hearing loss and 2 patients (Age 27 and 32) had marked bilateral conductive hearing loss on audiometry **Imaging** In the 2 patients with conductive hearing loss, lower HU values than the controls suggesting impaired bone mineralization **Histology** Transiliac crest biopsy revealed lower mean calcium content and a higher fraction of low mineralized bone areas compared to age-matched female controls Histomorphometric analysis indicated severe osteomalacia with higher osteoid volume per bone volume and bone volume to tissue volume compared to controls	Small sample size CCT scan and biopsy not performed in patients with sensorineural hearing loss Unclear current XLH treatment status Lack of clarification whether previous exposure to ototoxic drugs, previous ear surgery, chronic ear disease, and/or occupational exposure to noise were considered during recruitment
**Ivanovic-Zuvic et al./2021^14^**	Chile	*Cross-sectional* observational Patients recruited between May and November 2019 at the Center for Translational Research in Endocrinology of the Endocrinology Department, Pontificia Universidad Católica de Chile	*Human* Twenty-six XLH patients were confirmed on clinical, radiological, biochemical, and genetic analysis. Twenty adults and 6 children	Otolaryngologist evaluation (history/physical examination for symptoms and signs of hearing loss/Tinnitus/Vertigo) Audiometric evaluation: Tone audiometry, tympanometry and acoustic reflex performed by a medical technologist Full biochemical profile Full radiographic skeletal survey & calcaneus ultrasound	Otologic symptoms: Subjective hearing loss symptoms were reported in 12 adults (60%) and in no children **Audiometry** – Total of 6 (23%) confirmed hearing loss—4 adults (20%) and 2 children (33%) of ages 5 and 6 – Two sensorineural, 1 mixed, 1 conductive in adults – Two children both reported conductive hearing loss, showed signs of otitis media with effusion on physical examination – All 4 adults with hearing loss also reported episodic tinnitus – Lack of symmetry of severity and pattern of hearing loss between both ears in the same patient **Biochemical** – Lower serum phosphate in patients with hearing loss compared to those with no hearing loss **Other findings** – No significant differences in the treatment exposure and doses between patients with and without hearing loss – No significant association between otologic manifestations and the severity of other XLH manifestations	Small pediatric sample size Follow-up is required to monitor the trajectories of otitis media in pediatric patients—prevalent condition in all children Vestibular function evaluation and speech audiometry not performed
**Kato et al./2021^15^**	Japan	*Cross-sectional* observational Clinical records of patients who visited the University of Tokyo Hospital in 2015-2020 were retrospectively reviewed	*Human* Twenty-five XLH adults from 21 families. Thirteen males, 12 females. Median age 43 (range 18-72)	Pure-tone audiometry Clinical records on radiological and biochemical data and treatment status	**Audiometry** Hearing impairment was observed in 8 (32%) cases 5 males and 3 females Seven sensorineural, 1 mixed (53 year old male) All patients are being treated with either conventional therapy or burosumab No significant difference in the rate of hearing loss compared to previous studies with less proportion of patients on treatment Suggests lack of association between serum phosphate level and sensorineural hearing loss in XLH	Rudimentary—use of audiometry as the only otologic evaluation Audiometry only performed at a single time point in a patient Retrospective-unblinded evaluation Lack of childhood treatment records Potential overrepresentation of more severe cases as the recruitment center being one of the very few specialized XLH services available in Japan PHEX mutation not confirmed in 3 patients—due to rejection of mutation analysis These 3 cases were included as XLH due to compatible clinical presentations persistent since childhood, which were unlikely to be of an autosomal dominant or recessive hypophosphatemic phenotype One of them had a family history of XLH
**Chesher et al./2018^16^**	England	*Cross-sectional* observational Clinical records from a single adult inherited metabolic disease service were retrospectively reviewed	*Human* Fifty-nine XLH adults (19 males, 40 females) Age range 17-79	Audiometry	8 (14%): 4 males and 4 females had hearing impairment, increasing prevalence with age Predominantly sensorineural in nature—5 sensorineural, 3 mixed A 35 year old male had a bilateral moderate to severe sensorineural hearing loss evident from childhood requiring a hearing aid	Formal hearing assessment was only performed in those who reported subjective hearing problems. Audiometry raw data not shown Observations subjective to clinician bias Limited access to childhood records including treatments received Lack of direct control group
**Fishman et al./2004^17^**	USA	*Cross-sectional* observational Children’s Mercy Hospital and Clinics, Missouri, USA	*Human* 26 XLH patients – Sixteen XLH children (1-18 yr, median 9.0, 10 female, 6 male) – All of their 10 affected parents (22-55, 7 female, 3 male)	Pure-tone audiometry Tympanometry Stapedius reflex threshold	None of the children had hearing loss attributable to XLH Fifteen out of 16 children demonstrated normal hearing, 1 with bilateral profound hearing loss was found to have a coincidental Mondini congenital inner ear malformation on temporal bone CT Two adult males and 1 female (under 52) demonstrated sensorineural hearing loss (all with severe XLH-related orthopedic manifestations)	Short report—no raw otological assessment data presented Unclear current XLH treatment status
**Dahir et al./2022^18^**	USA	Single family *case series*	*Human* 5-generation single pedigree Twenty-two untreated persons with XLH Three children (2, 6, 17 yr) Nineteen adults	Presence of hearing loss or tinnitus was assessed via clinical notes, phone interviews, and clinic visits	40.9% reported tinnitus or hearing loss Became more common with age	No formal audiological testing performed, based on clinical reports Retrospective clinical notes review No investigations to look at cause of hearing loss Single family
**Yuan et al./2015^19^**	China	Single family *case series*	*Human* 4-generation single pedigree 4 persons with XLH 2 adults (33, 62 yo) 2 children (6, 13 yo)	Presence of hearing loss was assessed clinically with further investigations as clinically indicated	One case had bilateral mixed hearing loss (13 year old female) One had sensorineural hearing loss and mixed (62 year old female) One had no hearing loss (33 year old female) One not known (6 year old male)	No formal audiological testing performed, based on clinical reports Retrospective clinical notes review Small sample size Single family
**Popowska et al./2001^20^**	Poland	Single center *case series*: Department of Metabolic Diseases, Children’s Memorial Health Institute, Warszawa, Poland	*Human* Fifty-nine persons with XLH from 36 unrelated families Forty-five children (6mth to 16) Fourteen untreated adults	Presence in hearing loss was assessed via retrospective review of clinical notes	Three children had sensorineural hearing loss <25% had audiological testing Hearing defect correlated with genetic mutations in the beginning fragment of the *PHEX* gene (exons 1-5).	Not all participants had audiological testing Retrospective clinical notes review
**Pantel et al./2009^21^**	Switzerland	Single *case report*	*Human* Single case of fluctuating hearing loss in XLH	Single case report only	55 year old male with fluctuating hearing with audiogram showing sensorineural hearing loss (mainly low and high frequencies) Temporary improvement was seen with steroid therapy Stabilization after 1 year	Single case report only, unable to draw any conclusions from this report alone

**Table 3:** Animal studies—key study characteristics, results, and limitations.

**Author(s)/Year (Reference)**	**Country of origin**	**Study design**	**Population**	**Methodology**	**Results**	**Limitations**
**Delsmann et al./2021^13^**	Germany	*Animal study* - *Phex* mutant mice	*Animal* *Hyp/*Y male mice & Wild type (WT) control male mice at different ages (3, 6, 12, and 24 wk) All mice from C57BL/6 (B6) background	Auditory Phenotype: – Auditory Brainstem Response^13^ – Distortion product otoacoustic emission (DPOAE) – 9 *Hyp* & 6 WT at 6 wk – High-resolution micro-computed tomography ($\mu$-CT) & electron imaging of the auditory ossicles (5 *Hyp* & 5 WT at 24 wk) Histology of the auditory ossicles & cochleae (5 *Hyp* & 5 WT at 24 wk) Developmental analysis of the ossicles in WT mice at 3, 6, 12, and 24 wk	Auditory Phenotype: – Predominant conductive hearing loss in *Hyp* mice compared to WT mice. (elevated DPOAE/ ABR threshold shifts at mid-frequency regions) – Evidence of ELH in *Hyp* mice compared to WT mice Imaging: – Severe hypomineralisation of the stapes and malleus in *Hyp* mice compared to WT – Significantly higher porosity (larger voids), lower mean calcium content, lower osteocyte lacunae number, and larger osteocyte area Histology: – Markedly high osteoid volume per bone volume in *Hyp* mice compared to WT, consistent with osteomalacia – Similar bone volume per tissue volume compared to WT – Voids in *Hyp* mice correspond to unmineralized osteoid – Mild ELH (Grade 1) detected in the cochleae of *Hyp* mice, none in WT. This suggests a mild sensorineural component Postnatal development of the ossicles in WT mice: – Voids present in malleus at 3 wk correspond to blood vessels – Rapid mineralization by 6 wk with a significant decrease in porosity – Regression of blood vessels replaced by osteoblast-laid bone matrix By 24 wk, a significant increase in mean calcium content and a decrease in osteocyte lacunae number and area. Small blood vessels and areas of osteoid	Male mice only Short life span (2 yr) of mice models compared to humans Small sample size Cochlear function and skeletal analysis in one age group only Nil therapeutic interventions (as opposed to human studies where recruited patients are commonly already on treatment) Decline in osteocyte lacunae number in the ossicles of *Hyp* mice could be due to aging process as shown in aging WT mice ELH observed in 5 *Hyp* mice could suggest mild sensorineural hearing loss, but no further cochlear assessments performed
**Wick et al./2017^22^**	USA	*Animal study* - *Phex* mutant mice	*Animal* Four males *Phex*^Hyp-Duk^/Y cohorts: 1) **WT control** (*n* = 16) 2) ***Phex***^**Hyp-Duk**^**/Y control** (*n* = 15) 3) *Phex*^Hyp-Duk^/Y **Prevention** (*n* = 6) - Phosphorus and calcitriol supplementation P7-40 4) *Phex*^Hyp-Duk^/Y **Rescue** (*n* = 7) Phosphorus and calcitriol supplementation P20-40 All mice from BALB/cUrd background	ABR threshold at P40 Serum analysis (phosphate, calcium, ALP, creatinine, FGF-23) at P40 Histological analysis of temporal bones and inner ear at P402	ABR: – Significantly better hearing in WT at all frequencies compared to all 3 *Phex* groups. No differences between *Phex* groups Histological analysis: – All 3 Phex groups had gross cochlear malformation and bones with soft, chalk-like consistency compared to WT. No differences between 3 Phex groups – Inner ear of Phex control showed severe ELH in all specimens and dysmorphic otic capsule with thickened osteoid that indicates poor mineralization compared to WT control – Two Phex treatment groups showed marked improvement in mineralization of otic capsule compared to Phex control, but still less organized than WT control. However, no evidence of improvement in ELH Serum analysis: – Significantly lower serum phosphate levels in *Phex* groups compared to WT. No statistically significant increase in treatment groups compared to Phex control – Significantly lower FGF-23 level in WT compared to all 3 Phex groups. 2 treatment groups had significantly higher FGF-23 level compared to Phex control – No significant difference in calcium levels between WT and Phex control and Phex prevention. Phex rescue had significantly lower calcium levels than WT and Phex control, but not the Phex prevention – Significantly lower ALP in WT compared to all 3 Phex groups – No significant differences in creatinine level between all 4 groups	Male mice only Small sample size and lack of normative data for serum analysis Potential underdosing in treatment groups Only used ABR threshold hence unable to distinguish between conductive and sensorineural hearing loss
**Megerian et al./2008^23^**	USA	*Animal study* - *Phex* mutant mice	*Animal* *Phex* ^Hyp-Duk^/Y mice & WT controls Two genetic background strains: – BALB/cUrd – C57BL/6 (B6)	ABR threshold analysis = (At postnatal days 21, 25, 30, 40, 60, 90, 131) Mutants ≥5 and WT ≥ 3 at each timepoint) Histological analysis of temporal bones and cochleae (At diff postnatal days; P0-P300). Mutants ≥5 and 2 WTs at each timepoint)	BALB/cUrd background mice: – Vestibular dysfunction observed around P15, balance dysfunction evident in majority of mutants beyond P30 ABR: – At P21, many *Hyp-Duk* mice reported mild-moderate unilateral hearing loss – By P25 & beyond, the majority of *Hyp-Duk* mice developed bilateral and asymmetric hearing loss, which was progressive with age (P21-90) Histological analysis: – No abnormalities in the cochlear duct at birth (P0) to P10 in *Hyp-Duk* mice compared to WT. – No signs of ELH at birth such as endolymphatic duct obstruction – At P25, thickening of the otic capsule with unmineralized bone. Mild ELH but no endolymphatic duct obstruction – Beyond P40, severe ELH and displaced cochlear duct, however, no endolymphatic duct obstruction seen throughout the course in both mutant and WT – By P90, degeneration of the Organ of Conti and spiral ganglia apparent in mutants, but hair cells preserved – Age-related effects observed at P300 in WT B6 background mice: – **ABR**: No significant hearing loss in mutants compared to WT (P21-P131) – Some at P61 had mild unilateral hearing loss, typically had otitis media – No apparent ELH or generation in the cochleae in mutants (P28-131) – Marked thickening and impaired mineralization of the otic capsule in both ears of the mutants (P28-131)	Male mice only 2 different mutant background genetic strains—background effects on hearing Only used ABR threshold hence unable to distinguish between conductive and sensorineural hearing loss
**Lorenz-Depiereux et al./2004^24^**	Germany/USA	*Animal study* - *Phex* mutant mice	*Animal* Male mice with 4 different Phex mutant genotypes & respective WT male controls – *Hyp*/Y male – *Phex^Hyp-Duk^*/Y male – *Phex^Hyp-2J^*/Y male – *Gy*/Y male Between the ages 4-42 wk Background strains: *Hyp* & *Hyp-2J*: C57BL/6 (B6) strain *Hyp-Duk*: BALB/cUrd strain *Gy*: B6EiC3SnF1-a/A strain	ABR threshold analysis (4-42 wk of age) 4 *Hyp* + 5 WT 20 *Hyp-Duk* + 11 WT 13 *Hyp-2J* + 5 WT 6 *Gy* + 3 WT Histological analysis of the cochlea 6 *Hyp-Duk* & 3 WT 5-mo-old	All 4 mice models clinically present with similar phenotypes including shortened tail, square trunk and hind legs, as well as hypophosphatemia, hypocalcemia and rachitic bone disease ABR thresholds: – Significantly higher average ABR thresholds in 3 mutant strains (all except *Hyp-2J*) compared to their respective WT, suggesting hearing impairment – Most severe impairment was observed in *Hyp-Duk* mice. This distinction compared to other mutants was suggested to be due to the presence of recessive genetic modifiers in the BALB/cUrd strain that aggravate hearing loss – Hearing impairment was not progressive with age (P27-298) Histological analysis: – Hyp-Duk: Cochlear cross-sections revealed thickened temporal bone with multiple nonmineralized areas and cochlear precipitates in the scala tympani (both Hyp-Duk & Hyp-2J) – Degeneration of the organ of Corti & spiral ganglia in hearing impaired mutant mice. This was absent in Hyp-2J mice (normal hearing) – Endolymph volume was also enlarged, with diminished scala vestibule, enlarged scala media, and displaced Reissner’s membrane	Strain genetic background effects across the 4 genotypes, eg, presence of genetic modifiers, may result in varying severity of hearing loss across the mutants Male mice only Only used ABR threshold hence unable to distinguish between conductive and sensorineural hearing loss

Nine human studies,^13–21^ including 3 case series^18–20^ and one case report,^21^ and 4 animal studies^13^^,^^22–24^ were included. Within the 9 human studies, a total sample size of 229 XLH patients was represented, consisting of 157 adults and 72 children. Five studies included both adult and pediatric XLH patients, while the other 4 studied adults exclusively. 

All 4 animal studies utilized male mutant *Phex* mice models with their respective age-matched male wildtype (WT) control mice.

### Hearing in XLH adults

Various methodologies were used to assess hearing in XLH patients across the studies. Five studies^13–17^ performed formal audiometry on all their samples. Tympanometry was performed by Ivanovic-Zuvic et al.^14^ and Fishman et al.,^17^ with the latter also testing stapedial reflex thresholds. Hearing impairment was noted in a total of 30 XLH patients, with 28 adults and 2 children. The prevalence of hearing impairment was 71%^13^, 23%,^14^ 32%,^15^ 14%^16^, and 15%^17^ of XLH adults across the 5 studies. Of the 28 adults with hearing impairment, 20 had sensorineural hearing loss, 3 had conductive hearing loss, and 5 had mixed hearing loss. Each individual study also consistently reported predominant sensorineural hearing loss in their XLH adult cohorts.

Delsmann et al.^13^ performed cranial CT scans of the auditory ossicles on 2 adults with severe bilateral conductive hearing loss and compared these findings to those of healthy controls. Scans of the XLH adults showed impaired mineralization and severe osteomalacia of the auditory ossicles. Transiliac crest biopsies also showed lower mean calcium content and higher fraction of low mineralized bone areas compared to the control group, indicating severe osteomalacia.

Of note, Ivanovic-Zuvic et al.^14^ reported that subjective hearing loss was reported in 12 adults, while only 4 had confirmed hearing loss. Episodic tinnitus and vertigo were commonly reported in both patients with and without confirmed hearing loss. All 4 adults with confirmed hearing loss reported episodic tinnitus; however, no significant association was observed between otologic manifestations and the severity of other XLH manifestations. There was also no symmetry of severity and pattern of hearing loss between either ear in a single patient.

Of the 20 adults included in this study, 8 (40%) were on conventional treatment with activated vitamin D and phosphate. Analysis of biochemical parameters showed that serum phosphate was lower in patients with hearing loss compared to those with no hearing loss, with no differences seen with other biochemical markers, treatment exposure rate, dosage regime, demographics, or imaging. Studies done on those on treatment, conventional or burosumab, showed similarly high prevalences of hearing impairment, including Kato et al.^15^ reporting a prevalence of 32% of hearing impairment despite all their patients being treated with either conventional therapy or burosumab. Another study by Ivanovic-Zuvic et al.,^14^ where 40% of adults were on conventional therapy demonstrated a prevalence of 20% of hearing impairment.

### Hearing in XLH children

Hearing impairment was found only in 2 children with XLH, reported in the study by Ivanovic-Zuvic et al.,^14^ which recruited only 6 children in total. They were 5 and 6 yr old and both were found to have conductive hearing loss and both showed signs of otitis media with effusion, a common condition in the general pediatric population. Neither of these children reported subjective hearing loss. All children were on conventional therapy with activated vitamin D, such as calcitriol and phosphate supplementation. Fishman et al.^17^ reported no hearing loss in 16 of its pediatric patients, which could be attributable to XLH. One child had a profound bilateral hearing loss, but this was due to a coincidental Mondini congenital inner ear malformation on temporal bone CT.

### Auditory phenotypes in Phex mutant mice

A wide range of methods was utilized across the studies, including ABRs, DPOAEs, imaging and histology of the cochleae, temporal bones and auditory ossicles. ABR threshold analysis was performed in all 4 animal studies in the review.

Megerian et al.^23^ observed first onset of mild-moderate unilateral hearing loss in *Phex^Hyp-Duk^*/Y mice of BALB/cUrd background at Postnatal-Day-21 (P21). By P25 and beyond, most mice developed severe bilateral and asymmetric hearing loss, that was progressive with age (~P90). Vestibular dysfunction was also observed around P15 with balance dysfunction becoming prominent in most mutant mice by P30.

Studies however showed variable expression of auditory phenotypes between different background genetic strains of mutant mice. When the same *Hyp-Duk* mice from a different C57BL/6 (B6) background strain were studied, however, ABR threshold analysis showed no significant hearing impairment in mutant mice compared to WT from P21 to P131, with only some mutants with mild unilateral hearing loss at P61. This was also seen in the study by Lorenz-Depiereux et al.,^24^ which studied 4 groups of mutant mice, each with 4 different *PHEX* genetic alleles. *Hyp* and *Hyp-2J* mice were from B6 strain, *Hyp-Duk* from BALB/cUrd strain, and *Gy* from B6EiC3SnF1-a/A strain. All 4 groups clinically presented very similar phenotypes of shortened tail, trunk, and hind legs, with hypophosphatemia, hypocalcaemia, and rachitic bone disease. All 4 mutations have been found to cause a complete loss of *PHEX* function. On ABR analysis, however, despite all groups except *Hyp-2J* exhibiting significant hearing impairment compared to their respective WT, the hearing of *Hyp-Duk* mice from the BALB/cUrd strain was significantly more impaired compared to mutants from different a background strain. The study further analyzed the effects of background strain effects with inter-strain breeding of *Hyp-Duk* mice, which resulted in an attenuated hearing impairment that was then comparable to *Hyp* and *Gy* mutant mice. This indicated the presence of recessive genetic modifiers in the BALB/cUrd strain that exacerbate the auditory phenotypes in *Phex* mutant mice, supporting the findings by Megerian et al.^23^

### Pathological changes in the auditory ossicles and the cochleae in Phex mutant mice

Histological analysis of the ossicles and/or the inner ears was conducted in all 4 studies.

Normal postnatal development of the ossicles in the WT mice (B6 strain) was observed by Delsmann et al.,^13^ which showed voids in the malleus with blood vessels at 3 wk, which then rapidly mineralized by 6 wk as blood vessels regressed and were replaced with osteoblast-laid bone matrix. By 24 wk, a significant decrease in areas of osteoid and increase in mean calcium content was noted. This contrasted with the prominent osteomalacia of the ossicles in 24-wk-old mutant *Hyp* mice (B6 strain) with a markedly high osteoid volume per bone volume. A mild degree of ELH was detected in the cochleae of *Hyp* mice. High-resolution CT and electron imaging of the ossicles supported this with significantly higher porosity and lower mean calcium content demonstrating severe hypomineralisation. Histology of the temporal bones and the cochleae in *Hyp-Duk* mice at different ages by Megerian et al.^23^ provided a detailed picture of the progressive pathological changes in the ears of *Phex* mutants.

BALB/cUrd *Hyp-Duk* mice showed no cochlear abnormalities or signs of ELH at birth. At P25, thickening and poor mineralization of the otic capsule was observed with mild ELH, closely following the onset of unilateral hearing loss at P21 noted on ABR analysis. Beyond P40, severe ELH was noted with displacement of the cochlear duct and by P90, degenerative changes were seen in the spiral ganglia and the Organ of Conti. Interestingly, despite severe ELH, no endolymphatic duct obstruction was observed throughout the course in both mutants and WT.

B6 *Hyp-Duk* mice, which had no significant hearing impairment on ABR compared to WT, showed marked thickening and impaired mineralization of the otic capsule at 4 mo. However, no apparent ELH or other degenerative changes in the cochleae were observed, in contrast to the BALB/cUrd *Hyp-Duk* mice, which developed progressive hearing loss. A few B6 *Hyp-Duk* mice with mild unilateral hearing loss at P61 typically had otitis media. This suggests susceptibility to otitis media may aggravate hearing loss, although to a minimal degree.

Comparison of hearing-impaired *Hyp-Duk* variant and normal-hearing *Hyp-2J* variant of the same background strain at 5 mo by Lorenz-Depiereux et al.^24^ further supports the role of ELH in pathogenesis. Both groups had thickened temporal bones with areas of poor mineralization. However, the degenerative changes of the organ of Conti and the spiral ganglia in the hearing-impaired *Hyp-Duk* mice were absent in normal-hearing *Hyp-2J* mice. Enlarged endolymph volume was also noted in hearing-impaired *Hyp-Duk* mice, evident through displaced Reissner’s membrane, diminished scala vestibule, and enlarged scala media. The authors suggest this increase in endolymph volume as the likely cause of sensorineural degeneration and subsequent hearing impairment.

### Therapeutic interventions in Phex mutant mice

Wick et al.^22^ added further insight into the findings described above by investigating the effects of supplemental phosphate and calcitriol on hearing in BALB/cUrd *Phex*^Hyp-Duk^/Y mice. Four cohorts were studied: WT, *Hyp-Duk* control, *Phex* prevention diet, and *Phex* rescue diet. The *Phex* prevention cohort received supplementation between P7 and P40, while the rescue cohort received supplementation between P20 and P40. ABR analysis showed significantly better hearing in WT at all frequencies compared to all 3 *Phex* cohorts. There was no significant difference across the 3 *Phex* cohorts.

Histologically, all 3 *Phex* groups showed gross cochlear malformation compared to WT, but no significant differences were present within the 3 *Phex* groups. However, both rescue and prevention groups showed marked improvement in the mineralization of the otic capsule compared to the *Hyp-Duk* control group, although they did not reach the full organization level of the WT control. Of note, there was no evidence of improvement in the degree of ELH in rescue and prevention cohorts despite supplementation. In this study, serum phosphate levels were also significantly lower in the 3 *Phex* groups compared to WT, but no statistically significant deviation within the 3 *Phex* cohorts. This may reflect inadequate dosing or timing of treatment, which requires further detailed investigation.

## Discussion

This review summarizes the limited literature exploring hearing loss in XLH. The variable methodologies and study designs, while a limitation of this review, were also a strength, as each study presented unique insights into pathogenesis and implications of hearing loss in this population. The paucity of adequate patient studies is another limitation of this review. The few studies meeting the inclusion criteria were also limited in that most were cross-sectional with no longitudinal follow-up. Therefore, the natural pathogenesis of hearing impairment could not be determined. Incomplete assessment and lack of formal audiometry were also a limiting factor and likely under-reports the true prevalence of hearing impairment in XLH. There were also various inconsistencies across the studies, which meant drawing definitive conclusions was not possible. However, these findings highlight several areas for future research. One unanswered question is whether the introduction of burosumab will impact the development of hearing loss in individuals with hearing loss. Therefore, longitudinal studies looking at the impact of burosumab should include an assessment of hearing loss if possible.

### Hearing loss in XLH in adults and children

While methodologies were variable across only a small number of studies, the reported studies suggest that hearing impairment is more prevalent in adults with XLH than in the general population, with prevalences ranging from 14%-71%. This is generally higher than the global prevalence of hearing impairment of 6.8%-14.1% in adults from the general population.^14^ The majority of hearing loss in adults with XLH was noted to be sensorineural.

The prevalence of younger adult patients with hearing loss was notably higher in adults with XLH compared to the general population.

Hearing loss is a common chronic condition that increases with age and is influenced by both genetic and environmental factors. Prevalence varies across studies due to genetic and socioeconomic differences, as well as nonstandardized methods of epidemiologic data collection.^25^ However, cross-sectional national data from the USA (1999-2018) indicate that hearing loss prevalence is less than 10% among individuals aged 20-29 and 30-39, but rises sharply to at least 30% in those aged 60-69.^25^^,^^26^ A similar trend is observed globally, as reported in the Global Burden of Disease 2019 study.^27^ The studies included in our review examined XLH patients across a broad age range (17-79 yr). Four^13–15^^,^^17^ of the 5 studies provided age ranges for a total of 20 hearing-impaired XLH patients, all of whom were under 64 yr old. Additionally, 2 studies^13^^,^^15^ provided raw demographic data, showing that 4 out of 6 (67%) and 4 out of 10 (40%) XLH adults between 20 and 39 yr old were hearing impaired. This reported prevalence is substantially higher than those observed in the general population for this age group (less than 10%), suggesting that hearing impairment may develop earlier in XLH patients compared to the general population.

While the pediatric studies looking specifically at hearing loss in XLH were small,^14^^,^^17^ and therefore do not allow any definitive conclusions to be drawn, they did not suggest that hearing impairment in children with XLH was more common than in the general population. When it does occur, it appears to be unrelated to the underlying condition and more likely due to common childhood conditions such as otitis media with effusion.

Looking beyond these cross-sectional studies, there are a number of case series describing single-family pedigrees who did not all undergo systematic formal audiological testing, reporting hearing loss or tinnitus in over 40% of the 22 affected family members^18^ or mixed/sensorineural hearing loss in at least 2 out of 4 affected family members.^19^ A single center case series of 59 people from 36 unrelated families reported sensorineural hearing loss in at least 3 children^20^ but, again, not all participants underwent audiological testing. These findings highlight the scarcity of data in this area but suggest that clinicians should consider formal audiological testing for all adults with XLH. For children, there is not enough evidence to suggest the need for routine audiological testing. Clinical guidelines are variable in what they recommend; Haffner et al.^4^ suggest an initial assessment once the audiological testing is feasible (at around 5 yr of age), with hearing evaluation sought if there are symptoms from 8 yr of age into adulthood.

### Hearing loss in Phex mutant mouse models

Despite the variable expression of auditory phenotypes and cochlear pathologies between different background genetic strains, the findings from the 4 animal studies collectively suggest ELH as the main cochlear pathology responsible for hearing impairment in *Phex* mutant mice. A wide range of methods were utilized in *Phex* mutant mice studies, with each providing unique insights into the pathogenesis of hearing loss in XLH. The findings demonstrate that hearing is affected from a very young stage of life in *Hyp-Duk* mice but that it only develops hearing loss postnatally.^23^ The background genetic strain may have a significant effect on the auditory phenotypes in *Phex* mutant mice, with different strains of Phex mutant mice demonstrating different hearing phenotypes.^14^^,^^23^

Histological analysis demonstrated prominent osteomalacia of auditory ossicles across *Phex* mutant mice regardless of background genetic strains. There was also evidence that functional degeneration may precede structural degeneration.^23^ Presence of ELH in hearing-impaired *Hyp-Duk* (BALB/cUrd) mice but absence in hearing-preserved *Hyp-Duk* (B6) mice highlights the crucial role of ELH in the pathogenesis of hearing loss in XLH and that the bone abnormality alone cannot cause hearing loss. Interestingly, in the analysis by Megerian et al.,^23^ ELH was seen without endolymphatic duct obstruction, suggesting that endolymphatic duct obstruction is not a prerequisite for the pathogenesis of ELH. The presence of otitis media in a small number of B6 *Hyp-Duk* mice also suggests that a susceptibility to otitis media may also aggravate hearing loss, something that may evidently also impact humans with XLH.

### Effect of XLH treatments on hearing loss

Lower serum phosphate levels were associated with hearing loss in 2 studies: 1 human^14^ and 1 animal study.^22^ In the clinical study by Ivanovic-Zuvic et al.,^14^ there was no association between prevalence of hearing loss and other biochemical markers, including those that better indicate longer-term phosphate levels (such as alkaline phosphatase). Furthermore, the treatment exposure rate/dosage regimen, demographics, and imaging between patients with and without hearing loss also showed no significant differences. This suggests either limited benefits of conventional therapy in hearing loss or may be a reflection of the small sample size. The other reviewed studies presented no evidence that treatment of XLH impacted incidence or severity of hearing impairment. Studies looking at treated populations did not show significantly different prevalences of hearing loss than those with only a fraction of their patients on treatment.^14^^,^^15^

This is consistent with findings from Wick et al.,^22^ who looked at the effect of supplemental phosphate and calcitriol on *Phex* mutant mouse models. This study demonstrated that conventional XLH therapy may alleviate hypomineralisation, hence the conductive component of hearing loss, but does not necessarily improve the severity of ELH or the overall hearing. Together, these findings, while based on small studies with limited subjects, highlight the need to consider hearing assessments in those with XLH, regardless of therapy or severity of disease.

Wick et al.^22^ also demonstrated that rescue and prevention cohorts had significantly higher serum FGF-23 levels compared to *Hyp-Duk* control cohort, reflecting the expected limitation of conventional therapy in XLH. Direct FGF23 inhibition with burosumab may induce greater improvement in bone mineralization, potentially to the extent that affects the degree of ELH and overall hearing. Hence, this may be a potential target for future studies using *Phex* mutant mice.^22^ There were no clinical studies looking at the impact of burosumab on hearing loss. In addition, a recent article summarizing the opinion of a group of 8 expert clinicians on anticipated impacts of long-term burosumab reported a low level of agreement on the effect of burosumab on hearing loss in XLH.^28^ Future studies aiming to identify whether burosumab will reduce the risk of hearing loss in XLH should include regular monitoring of hearing with formal audiology in their long-term follow-up of patients.

### Clinical implications for individuals with XLH and future directions

As summarized in Table 1, historical studies prior to 2000 have noted low-frequency hearing impairment in XLH patients with audiometry and tympanic electrocochleogram suggestive of cochlear hearing loss with ELH as the main pathology, similar to that in Ménière’s disease.^6^^,^^7^ Petrous bones showed generalized osteosclerosis and thickening with narrowing of internal auditory meatus at its mid-point, compared to control groups.^8^ Despite our 5 human studies not performing targeted studies such as electrocochleography necessary to assess for the presence of ELH, results show predominant sensorineural hearing loss with vertigo and tinnitus frequently co-reported in hearing-impaired XLH patients, resembling Ménière’s disease.

The exact pathophysiology of ELH in XLH is unclear. Previously proposed mechanisms involved morphological changes through petrous bone osteomalacia, disrupting physiologic endolymphatic turnover via affecting endolymph production in the stria vascularis and/or causing obstruction of endolymphatic duct or sac.^8^ Evidence suggests that bone abnormality alone is not the sole factor responsible for marked hearing impairment in XLH nor endolymphatic duct obstruction. Early treatment may be crucial to alleviate the hypomineralisation of the auditory ossicles observed in both our animal and human studies, but there is limited evidence regarding the efficacy of conventional therapy in altering the pathogenesis of ELH and hearing impairment in XLH. Currently, management of hearing impairment in XLH is limited to supportive measures such as hearing aids or cochlear implants, prevention of noise exposure and avoidance of ototoxic drugs.

In Ménière’s disease, which also presents with ELH and sensorineural hearing loss as in XLH, a defective periductal interstitial connective tissue network has been proposed as the key factor that leads to disrupted homeostasis of the endolymphatic system contributing to the pathogenesis of ELH.^29^^,^^30^ Management of Meniere’s disease is complex and multimodal, from dietary to pharmacological and surgical interventions. Several studies have investigated the role of corticosteroids in treating Meniere’s disease and ELH. The evidence for the efficacy of oral corticosteroids in improving hearing impairment remains weak, with some improvements noted in vertigo but not in hearing.^31^^,^^32^ Pantel et al.^21^ reported a 3-yr audiological follow-up of a 55-yr-old man with untreated XLH, who initially presented with fluctuating bilateral hearing loss. Audiometry confirmed symmetrical moderate to severe sensorineural hearing loss in both low and high-frequency ranges. After a 2-wk course of oral corticosteroids, the patient showed marked but temporary improvement in low to medium frequencies, lasting for 4 wk. Hearing thresholds continued to fluctuate until stabilizing spontaneously at 1 yr. Intratympanic corticosteroids, another modality used in Meniere’s disease after failed first-line noninvasive therapy, have shown mixed results regarding hearing improvement in ELH.^33–35^ The effectiveness of management options for Ménière’s disease in treating hearing impairment in XLH remains to be elucidated and warrants further research.

Lower serum phosphate levels were noted in both hearing-impaired XLH patients^14^ and hearing-impaired *Phex^Hyp-Duk^*/Y mice,^22^ however, whether phosphate levels can be a reliable biochemical marker to monitor or predict hearing impairment in XLH requires further research. In addition, the utility of serum phosphate is limited due to the fluctuation of levels throughout the day. In these studies, markers suggestive of chronic hypophosphatemia, such as elevated alkaline phosphatase (ALP) or radiological rickets, did not show any correlation with the rate of hearing loss. Hearing impairment is likely part of the natural history of XLH in adults and given the significant impact on quality of life, close follow-up and hearing monitoring are recommended.

The existing literature does not suggest that hearing impairment occurs more frequently in children with XLH than in the general population, and there is wide variability regarding the prevalence, pattern, and onset of hearing impairment in children with XLH. It is unclear whether long-term treatment with conventional therapy or burosumab from early childhood can prevent or delay the onset of hearing loss in XLH. Despite the onset of hearing impairment seen at very early stages of life in *Phex* mutant mice, hearing loss from cross-sectional cohort studies was reported in only 2 children of ages 5 and 6 yr across the included human studies, both with asymptomatic conductive hearing loss. Otitis media with effusion was also observed on physical examination, which is very common in otherwise healthy children of that age and known to cause conductive hearing loss. Hence, whether this is an independent disease from XLH or a progression to ELH due to XLH is unclear, and longitudinal follow-up of larger numbers of affected children would be beneficial for future studies to monitor the trajectories of otitis media in XLH. Consequently, routine hearing assessment may not be justified in asymptomatic children with XLH below the age of 5 yr, and for any symptomatic children with XLH, other otological causes should be thoroughly investigated and excluded in the first instance.

## Conclusions

There is a clear association between sensorineural hearing loss and XLH in adulthood; however, the pattern of hearing loss in children with XLH varies widely across studies. Findings from *Phex* mice models are inconclusive with many confounding factors, but largely suggest ELH as the key pathology that contributes to hearing loss in XLH. Bone abnormality alone likely does not contribute to hearing impairment in XLH. There is limited evidence on the efficacy of conventional therapy and burosumab in the management of hearing impairment in XLH despite clear benefits in preserving the mineralization of the auditory ossicles and the optic capsule and remains to be determined in future studies.

## Data Availability

No new data were created or analysed during this study. Data sharing is not applicable to this article.

## References

[1] Beck-Nielsen SS, Mughal Z, Haffner D, et al. FGF23 and its role in X-linked hypophosphatemia-related morbidity. Orphanet J Rare Dis. 2019;14(1):58. 10.1186/s13023-019-1014-8 30808384 PMC6390548

[2] Baroncelli GI, Mora S. X-linked hypophosphatemic rickets: multisystemic disorder in children requiring multidisciplinary management. Front Endocrinol. 2021;12:688309. 10.3389/fendo.2021.688309 PMC8378329 34421819

[3] Bhattacharyya N, Chong WH, Gafni RI, Collins MT. Fibroblast growth factor 23: state of the field and future directions. Trends Endocrinol Metab. 2012;23(12):610–618. 10.1016/j.tem.2012.07.002 22921867 PMC3502714

[4] Haffner D, Emma F, Eastwood DM, et al. Clinical practice recommendations for the diagnosis and management of X-linked hypophosphataemia. Nat Rev Nephrol. 2019;15(7):435–455. 10.1038/s41581-019-0152-5 31068690 PMC7136170

[5] Sandy JL, Simm PJ, Biggin A, et al. Clinical practice guidelines for paediatric X-linked hypophosphataemia in the era of burosumab. J Paediatr Child Health. 2022;58(5):762–768. 10.1111/jpc.15976 35426466

[6] Davies M, Kane R, Valentine J. Impaired hearing in X-linked hypophosphataemic (vitamin-D-resistant) osteomalacia. Ann Intern Med. 1984;100(2):230–232. 10.7326/0003-4819-100-2-230 6691666

[7] O'Malley S, Ramsden RT, Latif A, Kane R, Davies M. Electrocochleographic changes in the hearing loss associated with X-linked hypophosphataemic osteomalacia. Acta Otolaryngol. 1985;100(1–2):13–18. 10.3109/00016488509108581 4040696

[8] O'Malley SP, Adams JE, Davies M, Ramsden RT. The petrous temporal bone and deafness in X-linked hypophosphataemic osteomalacia. Clin Radiol. 1988;39(5):528–530. 10.1016/S0009-9260(88)80224-1 3180671

[9] Boneh A, Reade TM, Scriver CR, Rishikof E, Opitz JM, Reynolds JF. Audiometric evidence for two forms of X-linked hypophosphatemia in humans, apparent counterparts of Hyp and Gy mutations in mouse. Am J Med Genet. 1987;27(4):997–1003. 10.1002/ajmg.1320270434 3425609

[10] Davies RA . Chapter 11: Audiometry and other hearing tests. In: Furman JM, Lempert T, eds. Handbook of Clinical Neurology. Vol. 137. Elsevier; 2016:157–176. 10.1016/B978-0-444-63437-5.00011-X 27638069

[11] Meisler M . Mutation watch: PEX PLUS? Gene(s) for X-linked hypophosphatemia and deafness. Mamm Genome. 1997;8(8):543–544. 10.1007/s003359900499 9250856

[12] Piret SE, Thakker RV. Mouse models for inherited endocrine and metabolic disorders. J Endocrinol. 2011;211(3):211–230. 10.1530/JOE-11-0193 21765099

[13] Delsmann MM, Seist R, Stuerznickel J, et al. Conductive hearing loss in the Hyp mouse model of X-linked hypophosphatemia is accompanied by hypomineralization of the auditory ossicles. J Bone Miner Res. 2021;36(12):2317–2328. 10.1002/jbmr.4443 34523743 PMC8688200

[14] Ivanovic-Zuvic D, Santander MJ, Jimenez M, Novoa I, Winter M, Florenzano P. Characterization of otologic involvement in patients with X-linked hypophosphatemia. Clin Otolaryngol. 2021;46(6):1251–1256. 10.1111/coa.13825 34170626

[15] Kato H, Koga M, Kinoshita Y, et al. Incidence of complications in 25 adult patients with X-linked hypophosphatemia. J Clin Endocrinol Metab. 2021;106(9):E3682–e3692. 10.1210/clinem/dgab282 33912912

[16] Chesher D, Oddy M, Darbar U, et al. Outcome of adult patients with X-linked hypophosphatemia caused by PHEX gene mutations. J Inherit Metab Dis. 2018;41(5):865–876. 10.1007/s10545-018-0147-6 29460029 PMC6133187

[17] Fishman G, Miller-Hansen D, Jacobsen C, Singhal VK, Alon US. Hearing impairment in familial X-linked hypophosphatemic rickets. Eur J Pediatr. 2004;163(10):622–623. 10.1007/s00431-004-1504-z 15290264

[18] Dahir KM, Black M, Gottesman GS, et al. X-linked hypophosphatemia caused by the prevailing north American PHEX variant c.^*^231A>G; exon 13-15 duplication is often misdiagnosed as ankylosing spondylitis and manifests in both men and women. JBMR Plus. 2022;6(12):e10692. 10.1002/jbm4.10692 36530187 PMC9751662

[19] Yuan L, Wu S, Xu H, et al. Identification of a novel PHEX mutation in a Chinese family with X-linked hypophosphatemic rickets using exome sequencing. Biol Chem. 2015;396(1):27–33. 10.1515/hsz-2014-0187 25060345

[20] Popowska E, Pronicka E, Sułek A, et al. X-linked hypophosphatemia in polish patients. 2. Analysis of clinical features and genotype-phenotype correlation. J Appl Genet. 2001;42(1):73–88. 14564066

[21] Pantel G, Probst R, Podvinec M, Grtler N. Hearing loss and fluctuating hearing levels in X-linked hypophosphataemic osteomalacia. J Laryngol Otol. 2009;123(1):136–140. 10.1017/S0022215107001636 18279571

[22] Wick CC, Lin SJ, Yu H, Megerian CA, Zheng QY. Treatment of ear and bone disease in the Phex mouse mutant with dietary supplementation. Am J Otolaryngol. 2017;38(1):44–51. 10.1016/j.amjoto.2016.09.014 27733274 PMC6221453

[23] Megerian CA, Semaan MT, Aftab S, et al. A mouse model with postnatal endolymphatic hydrops and hearing loss. Hear Res. 2008;237(1–2):90–105. 10.1016/j.heares.2008.01.002 18289812 PMC2858221

[24] Lorenz-Depiereux B, Guido VE, Johnson KR, et al. New intragenic deletions in the Phex gene clarify X-linked hypophosphatemia-related abnormalities in mice. Mamm Genome. 2004;15(3):151–161. 10.1007/s00335-003-2310-z 15029877 PMC2859190

[25] Sheffield AM, Smith RJH. The epidemiology of deafness. Cold Spring Harb Perspect Med. 2019;9(9). 10.1101/cshperspect.a033258 PMC6719589 30249598

[26] Mo F, Zhu S, Jia H, et al. Trends in prevalence of hearing loss in adults in the USA 1999-2018: a cross-sectional study. BMC Public Health. 2024;24(1):976. 10.1186/s12889-024-18426-9 38589845 PMC11000291

[27] Hearing loss prevalence and years lived with disability, 1990-2019: findings from the global burden of disease study 2019. Lancet. 2021;397(10278):996–1009. 10.1016/S0140-6736(21)00516-X 33714390 PMC7960691

[28] Seefried L, Duplan MB, Briot K, et al. Anticipated effects of burosumab treatment on long-term clinical sequelae in XLH: expert perspectives. Front Endocrinol (Lausanne). 2023;14:1211426. 10.3389/fendo.2023.1211426 37547321 PMC10400326

[29] Hultgård-Ekwall AK, Couloigner V, Rubin K, Rask-Andersen H. Network organization of interstitial connective tissue cells in the human endolymphatic duct. J Histochem Cytochem. 2003;51(11):1491–1500. 10.1177/002215540305101109 14566021 PMC3957558

[30] Friberg U, Rask-Andersen H. Vascular occlusion in the endolymphatic sac in Meniere's disease. Ann Otol Rhinol Laryngol. 2002;111(3 Pt 1):237–245. 11913684 10.1177/000348940211100308

[31] Fisher LM, Derebery MJ, Friedman RA. Oral steroid treatment for hearing improvement in Ménière's disease and endolymphatic hydrops. Otol Neurotol. 2012;33(9):1685–1691. 10.1097/MAO.0b013e31826dba83 23047260

[32] Morales-Luckie E, Cornejo-Suarez A, Zaragoza-Contreras MA, Gonzalez-Perez O. Oral administration of prednisone to control refractory vertigo in Ménière's disease: a pilot study. Otol Neurotol. 2005;26(5):1022–1026. 10.1097/01.mao.0000185057.81962.51 16151353

[33] Garduño-Anaya MA, De Toledo HC, Hinojosa-González R, Pane-Pianese C, Ríos-Castañeda LC. Dexamethasone inner ear perfusion by Intratympanic injection in unilateral Ménière’s disease: a two-year prospective, placebo-controlled, double-blind, randomized trial. Otolaryngol Head Neck Surg. 2005;133(2):285–294. 10.1016/j.otohns.2005.05.010 16087029

[34] Christopher LH, Wilkinson EP. Meniere's disease: medical management, rationale for vestibular preservation and suggested protocol in medical failure. Am J Otolaryngol. 2021;42(1):102817. 10.1016/j.amjoto.2020.102817 33202330

[35] Arriaga MA, Goldman S. Hearing results of intratympanic steroid treatment of endolymphatic hydrops. Laryngoscope. 1998;108(11):1682–1685. 10.1097/00005537-199811000-00017 9818826

